# Feeding increasing amounts of Asparagopsis armata decreases enteric methane yield without compromising production in lactating dairy cows

**DOI:** 10.3168/jdsc.2025-0895

**Published:** 2026-04-23

**Authors:** Ezequiel Armand, M. Soraya Salloum, Rubén Giménez, Nicolas DiLorenzo, Marcelo De León, Marcos G. Zenobi

**Affiliations:** 1 Facultad de Ciencias Agropecuarias, Universidad Nacional de Córdoba, X5000 Córdoba, Argentina; 2 Instituto Nacional de Tecnología Agropecuaria, Manfredi, X5988 Córdoba, Argentina; 3 North Florida Research and Education Center, Department of Animal Sciences, Institute of Food and Agricultural Sciences, University of Florida, Marianna, FL 32446

## Abstract

Enteric methane (CH_4_) is the primary greenhouse gas emitted by ruminants. The red seaweed *Asparagopsis armata* effectively mitigates CH_4_, though optimal inclusion rates remain uncertain. The objective was to evaluate the dose–response effects of increasing *A. armata* inclusion levels on CH_4_ emissions, animal performance, feeding behavior, and bromoform (CHBr_3_) transfer to milk. Four multiparous Holstein cows (684 ± 56 kg BW, 35 ± 2 kg/d milk yield, and 146 ± 12 DIM) were randomly assigned to a 4 × 4 Latin square design. Cows received 1 of 4 treatments consisting of dietary inclusion of *A. armata* at 0%, 0.15%, 0.30%, and 0.45% of diet DM. The *A. armata* used had a concentration of 3 mg of bromoform per gram of DM. Each 21-d period included 14 d of adaptation and 7 d of measurement, during which DMI, milk yield and composition, CHBr_3_ transfer, methane emissions, and feeding behavior were measured. Enteric CH_4_ was measured using a laser methane detector (Crowcon Laser Methane Mini, Tokyo Gas Engineering Co. Ltd., Tokyo, Japan). Cows were fed a TMR ad libitum (5%–10% refusals). Data were analyzed with the MIXED procedure of SAS. Feeding *A. armata* reduced CH_4_ production, yield, and intensity linearly by up to ∼25%. Dry matter intake, milk yield, ECM, and BW were unaffected. Milk protein remained unchanged, but fat declined linearly with *A. armata* inclusion. Meal duration decreased linearly, and no CHBr_3_ was detected in milk. *Asparagopsis armata* shows strong potential as a feed additive to mitigate enteric CH_4_ without compromising production. Further studies should assess long-term effects on milk fat concentration and feeding behavior.

## Graphical Abstract

**Figure:**
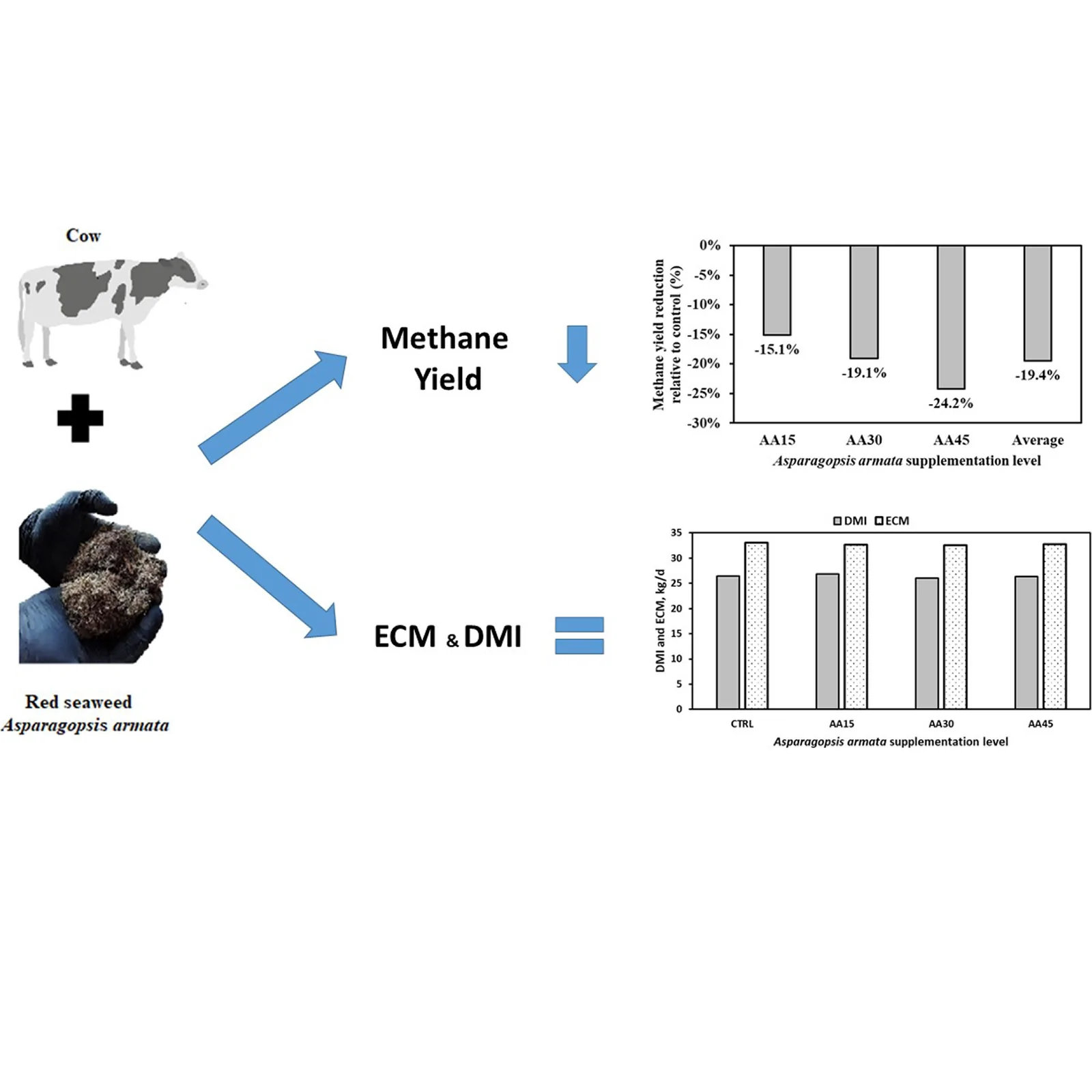

Ruminants are a major agricultural source of enteric methane, prompting research into mitigation strategies, including feed additives that inhibit methanogenesis. Red seaweeds of the genus *Asparagopsis* produce brominated compounds, primarily CHBr_3_, that inhibit methanogenic enzymes and consistently reduce enteric CH_4_ emissions in vivo (Genovese et al., 2012; Roque et al., 2019; Alvarez-Hess et al., 2023). However, higher inclusion levels may negatively affect dry matter intake and animal performance, and concerns remain regarding potential CHBr_3_ transfer to milk (Stefenoni et al., 2021). Previous studies have demonstrated effective methane mitigation at inclusion levels below 0.5% of diet DM, highlighting the importance of evaluating supplementation within this biologically relevant range. Therefore, the objective of this study was to evaluate the dose–response effects of increasing inclusion levels of *Asparagopsis armata*, within a range below 0.5% of diet DM, on enteric CH_4_ emissions, DMI, milk production, milk composition, and feeding behavior, and to assess potential CHBr_3_ transfer to milk. We hypothesized that increasing *A. armata* inclusion levels within the tested range would reduce enteric CH_4_ emissions without negatively affecting animal performance, milk composition, or feeding behavior, and would not result in detectable CHBr_3_ transfer to milk.

Animals were cared for according to the guidelines of The National University of Córdoba Institutional Animal Care and Use Committee (V-47/2023; CE-2023-00683819-UNC-SCT). Four multiparous Holstein cows (684 ± 56 kg BW; 35 ± 2 kg milk/d; 146 ± 12 DIM) were assigned to a 4 × 4 Latin square design (21-d periods: 14 d adaptation, 7 d sampling). Sampling included DMI and milk production (d 15–21), milk composition and CHBr_3_ (d 18), feeding behavior (d 20–21), and CH_4_ (d 17–20). Cows were individually penned with shade, feed bunks, and automatic waterers. Cows were randomly assigned to 1 of 4 treatments: control (**CTRL**; no *A. armata*) and dietary inclusion of *A. armata* at 0.15%, 0.30%, and 0.45% of diet DM (**AA15**, **AA30**, and **AA45**, respectively). The TMR (DM basis) contained 53.5% corn silage, 8.5% alfalfa hay, 15.0% ground corn, 21.5% soybean meal, and 1.5% mineral–vitamin mix. Diet composition (DM basis) was 16.1% CP, 28.0% NDF, 24.0% starch, 2.2% fatty acids, and 6.9% ash. The premix contained 1,350 mg/kg monensin on a DM basis. The *A. armata* product contained 3 mg of CHBr_3_/g DM. Four premixes were formulated by incorporating *A. armata* at concentrations designed to achieve final dietary inclusion rates of 0%, 0.15%, 0.30%, and 0.45% of diet DM, corresponding to the CTRL, AA15, AA30, and AA45 treatments, respectively. Target inclusion rates were achieved by replacing equivalent portions of the corn–soybean meal component of the premix with *A. armata*. Premixes were prepared at the beginning of each adaptation period and stored in a dry, dark environment until use. Each premix was mixed daily with the forage component to ensure accurate delivery of the intended *A. armata* concentration in the total diet. Based on a targeted DMI of 26 kg, supplementation levels of 0.15%, 0.30%, and 0.45% of diet DM corresponded to ∼39, 78, and 117 g *A. armata*/d, resulting in estimated CHBr_3_ intakes of 117, 234, and 351 mg/d, equivalent to 4.5, 9.0, and 13.5 mg CHBr_3_/kg DMI, respectively. Cows were housed individually in dry-lot pens (700 m^2^) equipped with J-bunks, fed ad libitum once daily at 0730 h (targeting 5% to 10% refusals), and had free access to water. All cows received the CTRL diet for 10 d before the start of the experiment for acclimation. The TMR met or exceeded NEL and MP requirements for a cow producing 35 kg/d of milk (3.50% fat, 3.05% true protein) and consuming 26 kg/d (NASEM, 2021). The basal TMR was prepared individually each day. Feed offered and refusals were weighed individually at feeding to determine daily intake. Samples of forages, concentrate, and refusals were collected 3 times weekly and dried for 72 h at 60°C for DM determination. Enteric CH_4_ emissions were measured during each collection period on 3 consecutive days at 4 time points relative to feeding (−1, 2, 8, and 12 h) using a laser methane detector (**LMD**; Crowcon Laser Methane Mini, Tokyo Gas Engineering Co. Ltd., Tokyo, Japan). Measurements followed Chagunda et al. (2009). Briefly, CH_4_ spot measurements were obtained at 0630, 0930, 1500, and 2100 h to represent daily variation. The timing and frequency of spot measurements were selected based on a previous field validation conducted under similar feeding and management conditions, which demonstrated that 4 measurements distributed across the day accurately captured daily methane emission patterns (Armand et al., 2022). Because meteorological conditions affect LMD readings (Ricci et al., 2014; Reintke et al., 2020), measurements were performed in wind-still conditions while considering temperature and humidity. To minimize artifacts from head movement, cows were measured in a cattle working chute without restraint. The operator maintained a fixed distance of 1 m from the nostrils. The LMD recorded CH_4_ concentrations at 0.5-s intervals for at least 5 min per cow. Values >3 SD from the overall mean were removed (Niero et al., 2020). The LMD outputs are expressed as ppm × m; therefore, total daily CH_4_ production (g/d) was estimated using the equation of Chagunda et al. (2009), which accounts for breath CH_4_ concentrations, activity duration, and tidal volume.

Dry matter intake was recorded daily (d 15 to 21) as feed offered minus refusals. Milk yield was recorded at each milking (d 15 to 21). Milk samples were collected on d 18 from 2 consecutive milkings (a.m. and p.m.). One aliquot was preserved with azidiol and analyzed for fat, true protein, and lactose by infrared spectrophotometry at a commercial laboratory (Laboratorio Villa María, Villa María, Cordoba, Argentina; ISO 9622:2013/IDF 141:2013; ISO and IDF, 2013). Composition values were weighted by milk yield. A second aliquot was frozen (−20°C) for CHBr_3_ determination, which was analyzed by static-headspace GC-MS (Farestaie Laboratories, Buenos Aires, Argentina) following Jordan et al. (2023). Limits of detection and quantification were 0.01 and 0.05 mg/L, respectively. Cow BW was recorded on 3 consecutive days (d 19–21). Feeding behavior was monitored for 2 d (d 20 and 21) in each period. Cows received their full ration at 0730 h. A single time-lapse camera (Brinno TLC 200 Pro, Taipei City, Taiwan) was positioned centrally to provide a complete and unobstructed view of the 4 individual pens. The camera captured images every 5 s for 12 h (0730–1930 h). Previously trained observers reviewed the images and classified cow activity at each time point as eating, drinking, standing, or lying based on head position and body posture, following procedures described in previous feeding behavior studies (Maltz et al., 2013). Eating was defined as the cow having its head positioned inside the feed bunk. The duration of each activity was calculated by summing consecutive images corresponding to each activity and multiplying by the sampling interval (5 s), and results were expressed in minutes and as a percentage of the 12-h observation period. Feeding bout frequency was defined as the number of distinct eating events during the observation period. A feeding bout was defined as a continuous sequence of images in which the cow was observed eating, separated from the next eating event by at least one image in which the cow was not eating. Meal duration was calculated as total eating time divided by the number of feeding bouts. Inter-meal interval was defined as the elapsed time between the end of one feeding bout and the beginning of the subsequent feeding bout. Enteric CH_4_ production (g/d), DMI, milk yield, BW, and feed efficiency (d 15 to 21) were averaged by cow and period. Enteric methane yield (g/kg DMI) and intensity (g/kg milk yield or g/kg ECM) were calculated as the ratio between CH_4_ production and intake or milk yield or ECM, respectively.

Data were analyzed using the MIXED procedure of SAS 9.4 (SAS Institute Inc., Cary, NC). The model included treatment and period as fixed effects and cow as a random effect. Treatment was evaluated as 4 inclusion levels of *A. armata* (0%, 0.15%, 0.30%, and 0.45% of diet DM), where the control diet represented the 0 inclusion level. Standard errors and denominator degrees of freedom were adjusted using the Kenward–Roger method. Normality of residuals was assessed using the Shapiro–Wilk test. Orthogonal contrasts were used to evaluate linear and quadratic responses to increasing *A. armata* inclusion. In addition, a planned contrast compared the control diet with the average of all *A. armata*-supplemented diets. Least squares means were generated using the LSMEANS statement in SAS, and pairwise comparisons among treatments were adjusted using the Bonferroni method. Data are presented as LSM ± SEM. Significance was declared at *P* < 0.05 and trends at 0.05 ≤ *P* ≤ 0.10.

The average CH_4_ production for each treatment is presented in Table 1 and illustrated in Figure 1. Compared with CTRL, dietary *A. armata* resulted in a linear reduction (*P* < 0.01) in enteric CH_4_ emissions of 15.1%, 19.1%, and 24.2% for AA15, AA30, and AA45, respectively (Figure 1). Methane yield (g CH_4_/kg DMI) and intensity (g CH_4_/kg milk yield or ECM) also declined linearly with increasing *A. armata* supplementation (*P* < 0.001).

**Table 1:** Dose–response effects of *Asparagopsis armata* supplementation on DMI, milk production, and enteric methane production, yield, and intensity in lactating dairy cows

Item	Treatment^1^				SEM^3^	*P-*value^2^		
	CTRL	AA15	AA30	AA45		CTRL vs. Trt	L	Q
DMI^4^ (kg/d)	26.5	26.8	26.0	26.3	0.67	0.65	0.09	0.81
Yield (kg/d)	31.6	31.4	31.2	32.0	0.81	0.96	0.72	0.49
ECM^5^ (kg/d)	33.0	32.6	32.6	32.7	0.82	0.69	0.79	0.75
Efficiency^6^ (kg/kg)	1.24	1.22	1.27	1.26	0.02	0.62	0.15	0.77
Fat								
%	3.72	3.66	3.67	3.55	0.11	0.08	0.02	0.42
kg/d	1.17	1.15	1.15	1.13	0.03	0.46	0.45	0.87
True protein								
%	3.47	3.48	3.51	3.45	0.08	0.78	0.70	0.21
kg/d	1.09	1.09	1.10	1.10	0.03	0.97	0.87	0.89
Lactose								
%	4.92	4.83	4.92	4.89	0.04	0.12	0.90	0.14
kg/d	1.55	1.52	1.53	1.57	0.05	0.75	0.70	0.35
BW^7^ (kg)	686	700	696	693	9.1	0.16	0.51	0.19
Enteric CH_4_ emissions								
CH_4_ (g/d)^8^	312	269	249	235	9.3	<0.01	<0.01	0.16
CH_4_ per DMI (g/kg)	11.8	10.0	9.6	9.0	0.48	<0.01	<0.01	0.20
CH_4_ per milk yield (g/kg)	9.9	8.7	7.9	7.4	0.26	<0.01	<0.01	0.23
CH_4_ per ECM yield (g/kg)	9.5	8.2	7.6	7.1	0.33	<0.01	<0.01	0.18

1 Treatments were control (CTRL) and *A. armata* supplemented at 0.15%, 0.30%, and 0.45% of diet DM (AA15, AA30, and AA45, respectively).

2 Contrasts: CTRL versus Trt = control versus all *A. armata* treatments; L = linear effect of *A. armata* supplementation level; Q = quadratic effect of *A. armata* supplementation level.

3 SEM = standard error of treatment means.

4 Data represent the average during the week of gas measurement.

5 ECM (kg/d) = [(0.3246 × milk, kg/d) + (12.86 × milk fat, kg/d) + (7.04 × milk protein, kg/d)] (Orth, 1992).

6 Feed efficiency = ECM yield/DMI.

7 BW = average body weight during the last 3 d of each period.

8 Methane emissions (g/d) were estimated from laser methane detector measurements using the equation described by Chagunda et al. (2009): CH_4_ (g/d) = 0.000576 × (ppm × m) × TV, where ppm × m represents the path-integrated methane concentration measured by the detector, TV is tidal volume (mL per breath), and tidal volume was assumed to be 3,800 mL for standing lactating dairy cows.

**Figure 1:**
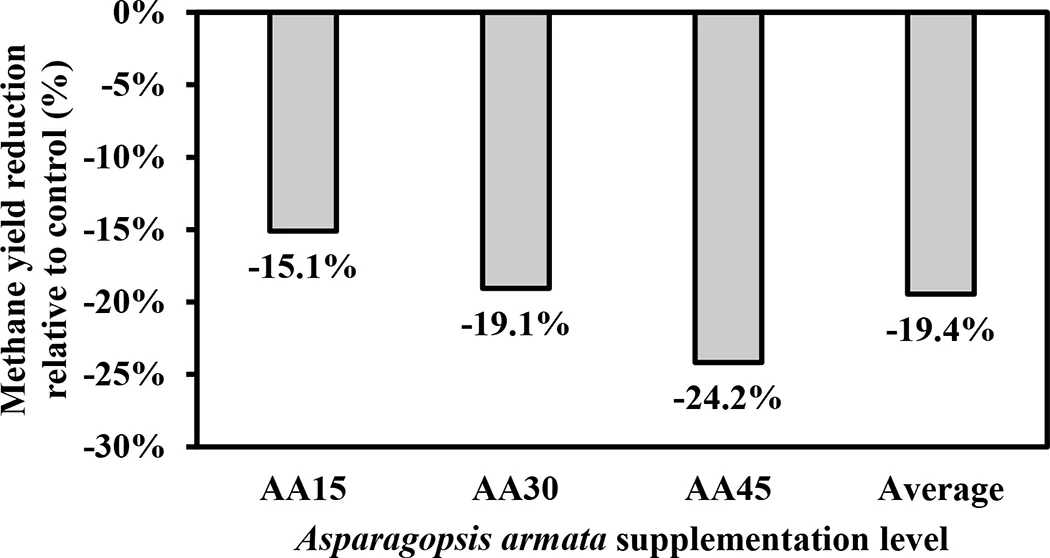
Reduction in methane yield in dairy cows supplemented with *Asparagopsis armata*. Treatments included a control diet and diets supplemented with *A. armata* at 0.15%, 0.30%, and 0.45% of diet DM (AA15, AA30, and AA45, respectively), corresponding to estimated CHBr_3_ intakes of 4.5, 9.0, and 13.5 mg/kg DMI, respectively. Methane yield was reduced by 15.1%, 19.1%, and 24.2% for treatments AA15, AA30, and AA45, respectively. Enteric methane emissions were measured using a laser methane detector (Laser Methane Mini, Tokyo Gas Engineering Co. Ltd., Tokyo, Japan). Values represent the percent reduction in methane yield (g CH_4_/kg DMI) relative to control. The average bar represents the mean methane yield reduction across *A. armata* treatments.

The effects of *A. armata* on BW, milk yield, and milk composition are summarized in Table 1. Dry matter intake averaged 26.4 kg/d, milk yield 31.5 kg/d, ECM 32.7 kg/d, and BW 694 kg, with milk total protein remaining constant at 3.48% across treatments. None of these variables differed among treatments (*P* > 0.10), except for milk fat concentration, which decreased linearly with increasing *A. armata* (*P* < 0.02). A weak linear tendency was observed for *A. armata* to reduce DMI (*P* = 0.09). Bromoform was not detected in milk samples from any treatment (limit of detection = 0.01 mg/L). This level is well below the established maximum permissible standard, indicating that *A. armata* inclusion at the tested doses did not result in measurable CHBr_3_ transfer to milk.

The effects of increasing *A. armata* on feeding behavior are summarized in Table 2. Time spent eating over a 12-h period decreased with higher *A. armata* inclusion, showing a linear trend (*P* = 0.05). Meal duration followed a similar pattern (*P* = 0.05), declining from 46.6 min in CTRL to 38.3 min in 45AA. In contrast, the number of eating bouts (*P* = 0.58) and inter-meal interval (*P* = 0.57) did not differ among treatments, indicating that cows spent less time eating but maintained meal frequency and spacing. These results suggest that higher *A. armata* doses influenced feeding patterns by shortening meals, potentially increasing meal size.

**Table 2:** Dose–response effects of *Asparagopsis armata* on feeding behavior in lactating dairy cows

Item	Treatment^1^				SEM^3^	*P* value^2^		
	CTRL	AA15	AA30	AA45		CTRL vs. Trt	L	Q
Eating (min/12 h)	269	242	230	221	15.8	0.06	0.05	0.53
Feeding bouts (events/12 h)	6.0	6.3	7.3	5.9	0.70	0.58	0.85	0.28
Meal duration (min/meal)	46.6	39.7	31.7	38.3	3.65	0.05	0.08	0.10
Inter-meal interval (min)	128	128	100	124	16.0	0.57	0.58	0.48
Drinking (min/12 h)	42.5	37.3	59.3	61.8	9.34	0.37	0.09	0.69
Standing (min/12 h)	135	120	128	146	24.0	0.90	0.71	0.51
Lying (min/12 h)	220	268	235	232	22.4	0.35	0.96	0.28
Eating (%)	40	36	35	33	2.49	0.10	0.09	0.63
Drinking (%)	6.4	5.7	9.1	9.5	1.48	0.33	0.08	0.73
Standing (%)	20	18	20	22	3.56	0.94	0.67	0.54
Lying (%)	33	40	36	35	3.22	0.31	0.90	0.25

1 Treatments were control (CTRL) and *A. armata* supplemented at 0.15%, 0.30%, and 0.45% of diet DM (AA15, AA30, and AA45, respectively).

2 Contrasts: CTRL versus Trt = control versus all *A. armata* treatments; L = linear effect of *A. armata* supplementation level; Q = quadratic effect of *A. armata* supplementation level.

3 SEM = standard error of treatment means.

Drinking behavior showed a tendency for cows receiving more *A. armata* to spend more time drinking (42.5 min in CTRL vs. 61.8 min in AA45). Although overall treatment effects were not significant (*P* = 0.37), a linear trend was detected (*P* = 0.09), suggesting that higher *A. armata* doses may increase water intake time.

This study evaluated the CH_4_-mitigating potential of *A. armata* in dairy cows, together with effects on productivity, feeding behavior, and bromoform transfer to milk. Our findings show that *A. armata* has a clear dose-dependent capacity to reduce enteric CH_4_ without detectable CHBr_3_ transfer to milk at the tested doses. However, milk fat percentage was reduced, potentially in association with altered feeding behavior. Although the present study included a limited number of cows, the use of a 4 × 4 Latin square with increasing doses of *A. armata* strengthens the robustness of the findings. In this design, each cow received all treatments and thus served as its own control, which reduces between-animal variation and increases statistical power despite the small sample size. In addition, the clear linear dose–response relationships observed for CH_4_ production, yield, and intensity across the inclusion levels provide internal consistency and biological plausibility, supporting the reliability of the treatment effects. Nonetheless, confirmation under commercial conditions and with larger number of animals and different statistical designs is warranted to assess the generalizability of these results.

Our results differ from several previous studies in which *Asparagopsis* spp. significantly reduced DMI in dairy cattle (Roque et al., 2019; Stefenoni et al., 2021). Roque et al. (2019) reported 11% and 38% reductions in DMI when 0.5% and 1.0% *A. armata* (OM basis) were included, whereas Stefenoni et al. (2021) observed more moderate decreases of 3% and 7% with 0.25% and 0.5% *Asparagopsis taxiformis* (DM basis). In addition, recent work by Eikanger et al. (2024) reported reductions in DMI and milk production at inclusion levels of *A. taxiformis* above 0.25%, further illustrating that negative intake and production responses can occur at low-moderate supplementation rates. In contrast, Kinley et al. (2020) found no negative effect on DMI with 0.05%, 0.10%, or 0.20% *A. taxiformis* (OM basis) in Brangus steers, and Roque et al. (2021) reported no effect at 0.25% *A. taxiformis* but a reduction in DMI at 0.5%. Differences among studies likely reflect diet, animal type, and seaweed CHBr_3_ content. In the present study, *A. armata* contained 3 mg CHBr_3_/g DM, approximately 2-fold higher than the 1.32 mg/g reported by Roque et al. (2019), but lower than the 6.55 and 7.80 mg/g DM reported for *A. taxiformis* by Roque et al. (2021) and Kinley et al. (2020), respectively. As in previous trials with *Asparagopsis* spp., the seaweed in our study was freeze-dried and incorporated into a TMR (Kinley et al., 2020; Roque et al., 2021; Stefenoni et al., 2021), a process shown to preserve bioactive compounds (Vucko et al., 2017). *Asparagopsis armata* did affect milk composition in this study, but only for a linear decrease in fat concentration (*P* < 0.02). To date, few studies have reported both CH_4_ and milk composition responses to *Asparagopsis* spp. in dairy cows. Roque et al. (2019) observed a decrease in milk protein only at 1% *A. armata* (OM basis), with no effects on other milk fractions at lower inclusion rates. Stefenoni et al. (2021) reported a reduction in milk lactose with 0.5% *A. taxiformis* (DM basis) but no changes in fat or protein. Taken together, the present and previous studies do not provide conclusive evidence regarding the effects of *Asparagopsis* spp. on milk composition, indicating that further work is needed.

*Asparagopsis armata* may have influenced feeding behavior by shortening meal duration and total eating time without changing DMI or bunk visit frequency, resulting in faster meals. Such shifts in feeding patterns could alter rumen fermentation, potentially changing the acetate:propionate ratio and favoring conditions associated with milk fat depression (**MFD**). The observed reduction in milk fat concentration might therefore be partially explained by altered fermentation, as faster meals can induce fluctuations in rumen pH and microbial activity that affect fat synthesis. Specific compounds in *A. armata* could also contribute directly to MFD, although mechanisms remain unclear. A depression in milk fat was also reported by Alvarez-Hess et al. (2023) with *A. armata* and by Muizelaar et al. (2021) with *A. taxiformis*, the latter also noting lower milk protein. In contrast, Stefenoni et al. (2021) observed no effect on milk fat or protein but did report lower lactose in cows fed *A. taxiformis*. These inconsistent findings highlight the need for further research on the impact of *Asparagopsis* spp. on milk composition and its underlying mechanisms.

Bromoform, a key bioactive compound in *Asparagopsis* spp., underpins its CH_4_-reducing effects in ruminant diets. As a natural defense mechanism, the macroalga produces CHBr_3_ together with other halogenated compounds, including dibromochloromethane, bromochloromethane, and dibromoacetic acid, which inhibit microbial growth (Paul et al., 2006; Machado et al., 2018). The concentration of these compounds varies with algal growth conditions (Shri Devi and Paul, 2014). Although CHBr_3_ is effective for CH_4_ mitigation, its potential toxicity to humans raises concerns. The US Environmental Protection Agency has set a reference dose for human oral exposure of 0.02 mg/kg BW per day, although limits for cattle have not been defined (EPA, 2017). Studies on CHBr_3_ transfer to milk have reported mixed outcomes: Roque et al. (2019) found no detectable CHBr_3_, whereas Stefenoni et al. (2021) observed small increases in CHBr_3_ and higher bromide and iodine levels when *Asparagopsis* was fed at 0.5% of dietary DM. In the present study, CHBr_3_ was not detected in milk samples (limit of detection = 0.01 mg/L), indicating no measurable transfer of CHBr_3_ to milk at the evaluated inclusion levels.

This study evaluated the potential of *A. armata* to reduce methane emissions in dairy cows without compromising milk yield or ECM. Feeding *A. armata* up to 0.45% of DMI reduced daily CH_4_ yield and intensity by ∼25%. Bromoform was not detected in milk samples, indicating no residue accumulation at the tested doses. A limitation of the present study is that only CHBr_3_ was quantified in milk samples. Other halogenated compounds potentially present in *A. armata* were not analyzed; therefore, conclusions regarding the absence of other brominated metabolites in milk cannot be drawn. Further research is warranted to evaluate the fate of these compounds in dairy production systems

However, a linear decrease in milk fat concentration was observed, potentially linked to changes in feeding behavior and ruminal fermentation. Although *A. armata* appears promising as a CH_4_-mitigating feed additive, further research is required to assess long-term effects on animal health, productivity, and milk composition before broad implementation in dairy systems.
